# Posterior Border Distance: An Effective Diagnostic Measurement for Carpal Tunnel Syndrome Using Ultrasonography

**DOI:** 10.7759/cureus.11010

**Published:** 2020-10-18

**Authors:** Gokhan Meric, Koray Başdelioğlu, Bahar Yanık, Serdar Sargin, Ali Engin Ulusal

**Affiliations:** 1 Orthopaedics and Traumatology, Yeditepe University, Istanbul, TUR; 2 Orthopaedics and Traumatology, Istanbul Oncology Hospital, Istanbul, TUR; 3 Radiology, Balıkesir University, Balıkesir, TUR; 4 Orthopaedics and Traumatology, Balikesir University, Balikesir, TUR; 5 Orthopaedics and Traumatology, Balikesir University, Balıkesir, TUR

**Keywords:** carpal tunnel syndrome, posterior border distance, ultrasonography, electrodiagnostic test

## Abstract

Purpose

The purpose of this study was to define posterior border distance (PBD), which represents an ultrasonographic diagnosing method of carpal tunnel syndrome (CTS), and to determine the reliability of PBD in comparison with electromyography (EMG) results.

Methods

Thirty-three patients (mean age: 51.8 ± 9.5 years; 27 females and six males) with CTS were included in this study. Ultrasonography (US) and EMG were performed under blinded conditions. PBD was evaluated by measuring the length of the perpendicular line between the posterior border of the median nerve and the line between the hook of the hamate and trapezoid tubercle. The cross-sectional area, anteroposterior (AP), and transverse diameter of the median nerve were measured. Control US was performed in 20 patients who were available at the first year postoperative follow-up and the results compared with preoperative US values. Correlation analyzes were performed to determine the relationship between electrodiagnostic results and ultrasonographic measurements.

Results

According to the results of preoperative and postoperative first-year US, there were statistically significant differences in the results of PBD (preoperative: 3.309±1.7472 mm, postoperative: 2.290±0.7867 mm p: 0.013) and AP diameter of the median nerve (preoperative: 3.012±0.7865 mm, postoperative: 2.680±0,5578 mm p: 0.017). There was no statistically significant difference in transverse diameter (preoperative: 6.585±1.9505 mm, postoperative: 6.955±2.2128 mm) and cross-sectional area (preoperative: 14.33±6.513 mm^2^, postoperative: 11.20±5.830 mm^2^) results (p>0.05).

The cut-off value of PBD was ≥3.6 mm, it yielded 81.48% specificity and 83.33% sensitivity in the diagnosis of CTS. PBD was correlated with motor and sensory latency, anteromedial, and transverse diameter of the median nerve (p<0.05). There was no correlation between EMG values and the results of the cross-sectional area, transverse diameter, and AP diameter of the median nerve (p>0.05).

Conclusion

PBD is suggested as a reliable ultrasonographic measurement method for the diagnosis of CTS.

## Introduction

Carpal tunnel syndrome (CTS) is the most common nerve entrapment neuropathy [1]. It is caused due to median nerve compression mostly beyond the distal wrist crease [2]. Mechanical trauma, increased pressure, and ischemic damage to the median nerve within the carpal tunnel constitute the pathophysiology of CTS [3].

The gold standard procedure for the diagnosis of CTS is controversial. The diagnosis of CTS is primarily based on clinical symptoms and often confirmed by an electrodiagnostic test [4]. However, 16% to 34% of patients may have false-negative electrodiagnostic results [5]. In addition, electrodiagnostic testing causes pain in patients [6].

Ultrasonography (US) has been demonstrated as a sensitive diagnostic method for CTS [6-8]. Many US parameters including median nerve flattening ratio, cross-sectional area of the median nerve at the carpal tunnel level, median nerve mobility, changes in nerve echogenicity and vascularity, and anteroposterior (AP) and transverse diameters of the median nerve have been described for confirming CTS diagnosis [9-12]. The measurement of the cross-sectional area of the median nerve has been accepted as the most predictive and reproducible method for the diagnosis of CTS [13,14]. Furthermore, the ultrasonographic diagnosis cutoff point, which has been used for the diagnosis of CTS, is highly variable, and there is no consensus reference standard for the diagnosis of CTS using US results. The purpose of this study was to define the posterior border distance (PBD), which represents an ultrasonographic method to diagnose CTS, and to determine the reliability of this method in comparison with electromyography (EMG) results.

## Materials and methods

The study included 33 patients (mean age: 51.8 ± 9.5 years, 27 females and six males) with the clinical evidence of CTS between 2013 and 2014. In our study, CTS was diagnosed based on two or more of the following criteria [5,15]: i) increased paresthesia confirmed by provocative tests (Phalen’s/Tinel’s signs), ii) nocturnal paresthesias, iii) increased pain and numbness while holding a book or phone, cycling, and driving, and iv) regression of symptoms by shaking hands.

Patients were subjected to US and EMG tests under single-blinded conditions. In EMG tests, a distal motor latency of ≥4.2 ms and/or a distal sensory latency of ≥3.2 ms was used as the cutoff point for a positive diagnosis of CTS [16].

**Figure 1:**
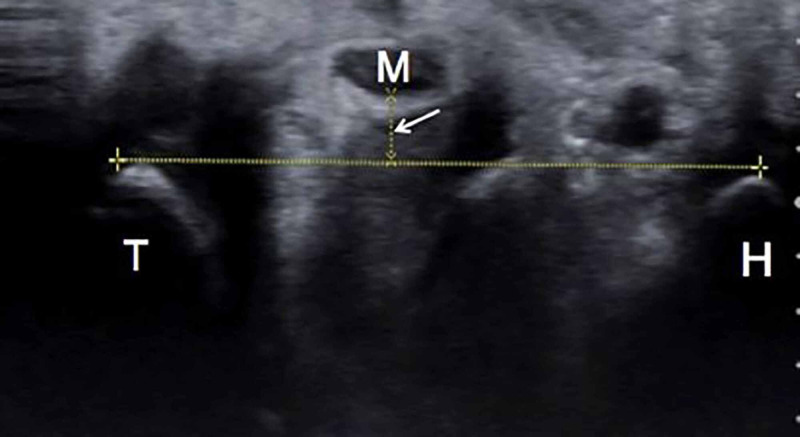
Posterior border distance Posterior border distance (as shown by the arrow) is the distance between the posterior border of the median nerve (M) and the line connecting the hook of the hamate (H) and trapezoid tubercle (T).

An experienced radiologist blinded to the patient’s history, as well as the results of physical examination and EMG tests performed using a Nicolet EDX (Natus, WI, USA), carried out all US tests using a 6-18 MHz multi-frequency linear array transducer (Toshiba Aplio MX SSA-780A, Tokyo, Japan). Patients were seated with the forearm supinated and the wrist and fingers in a neutral position. The ultrasound probe was positioned perpendicular to the long axis of the forearm, and the cross-sectional area, AP diameter, and transverse diameter of the median nerve were evaluated; AP and transvers diameters were measured at the hamate level. We evaluated the PBD by measuring the length of the perpendicular line between the posterior border of the median nerve and the line between the hook of the hamate and trapezoid tubercle (Figure 1). All measurements were repeated thrice, and median values were recorded. The study was approved by the ethical committee of our university.

Statistical analysis

All analyses were performed using Statistical Package for the Social Sciences (SPSS), version 22.0 (IMB Corp., Armonk, NY). Mean and standard deviation were used as descriptive statistical methods in the evaluation of data. The Mann-Whitney U test was used to compare quantitative continuous data between the two independent groups. The Spearman correlation test and regression analysis were performed to analyze continuous variables. The findings were evaluated at the 95% confidence interval and 5% significance level. Correlation analyses were performed to determine the relationship between EMG results and US measurements. Receiver operating characteristic (ROC) curve was drawn to evaluate the diagnostic value of the PBD.

## Results

Comparison between preoperative and postoperative US findings did not show any statistically significant differences in the transverse diameter (preoperative: 6.585 ± 1.9505 mm, postoperative: 6.955 ± 2.2128 mm, p > 0.05) and cross-sectional area (preoperative: 14.33 ± 6.513 mm2, postoperative: 11.20 ± 5.830 mm2, p > 0.05) of the median nerve. However, there were statistically significant differences in the PBD (preoperative: 3.309 ± 1.7472 mm, postoperative: 2.290 ± 0.7867 mm, p = 0.013) and AP diameter of the median nerve (preoperative: 3.012 ± 0.7865 mm, postoperative: 2.680 ± 0.5578 mm, p = 0.017) (Table 1).

**Table 1:** Preoperative and postoperative ultrasonography results

	Carpal Tunnel Syndrome (N:33)	Control (Postoperative 1st year) (N:20)	p	
	Mean±SD	Mean±SD		
Cross-Sectional Area	14.33±6.513	11.20±5.830	0.124	
Anteroposterior Diameter	3.012±0.7865	2.680±0,5578	0.017	
Transverse Diameter	6.585±1.9505	6.955±2.2128	0.752	
Posterior Border Distance	3.309±1.7472	2.290±0.7867	0.013	

In this study, the PBD was correlated with the motor and sensory latency as well as anteromedial and transverse diameter of the median nerve (p < 0.05). However, there was no correlation between the cross-sectional area, AP and transverse diameters of the median nerve, and EMG results.

Using a cutoff value of the PBD in ROC analysis, six positive and 27 negative values were obtained. Table 2 summarizes the results of the ROC analysis according to CTS index values.

**Table 2:** Receivers operating characteristic (ROC) curve data

Area under the ROC curve	0.796
Standard Error	0.125
95% Confidence interval	0.620 – 0.916
z statistic	2.378
Youden index	0.6481
Significance level of area (P=0.05)	0.0174

**Figure 2:**
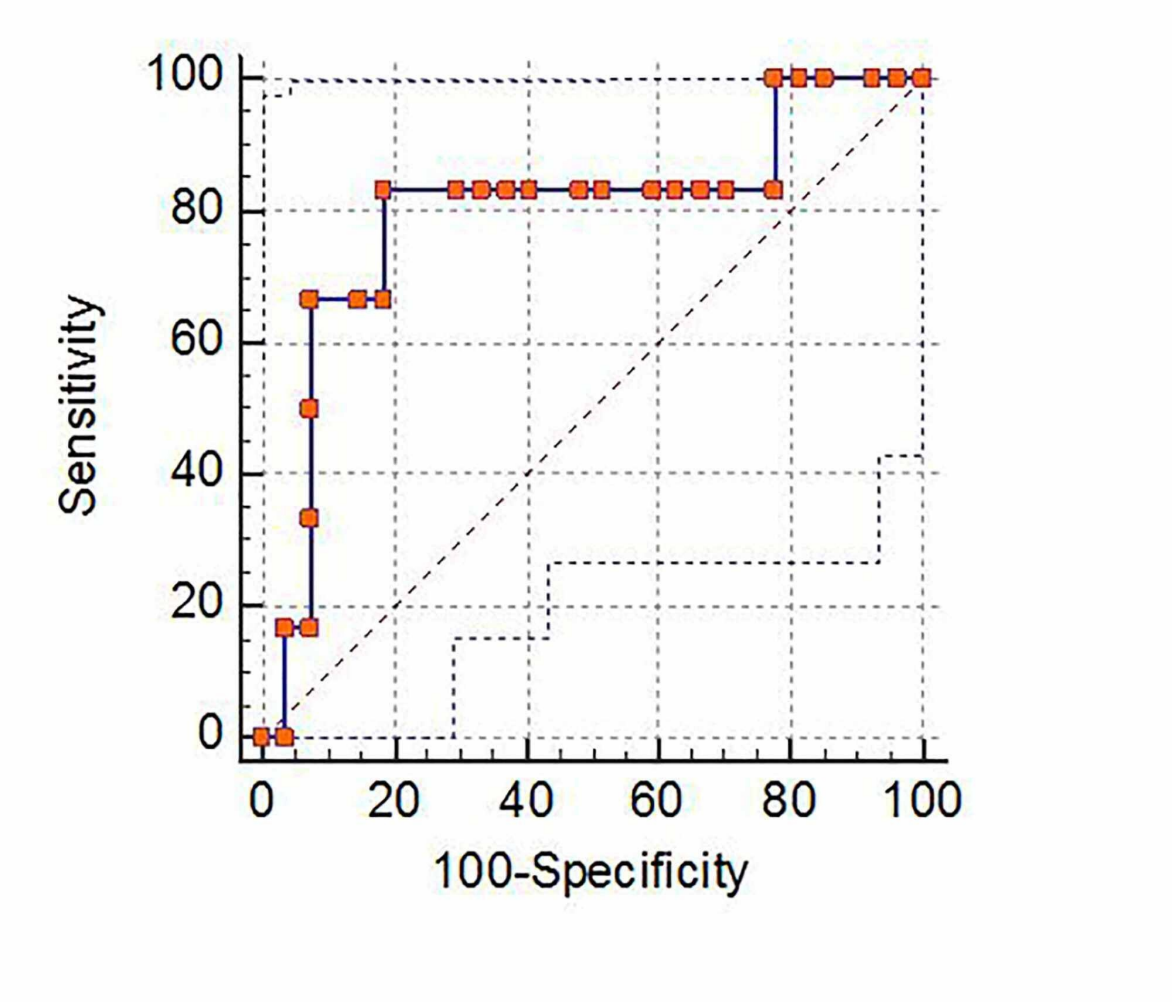
The area under the receiver operating characteristic (ROC) curve was statistically significant (p<0.05)

The optimum cutoff value of the PBD was determined to be >3.6 mm, which yielded 83.33% sensitivity and 81.48% specificity. The Youden index (J) was 0.6481 (0 < J = 0.6481 <1) (Figure 2).

## Discussion

In our study, the PBD showed correlation with electrodiagnostic parameters with high specificity and sensitivity. We recommend the ultrasonographic measurement of the PBD as a non-invasive and reliable method for the diagnosing of CTS. The median nerve shows palmary displacement in patients with CTS, and we measured the distance between the median nerve and the line between the hook of the hamate and trapezoid tubercle to define this movement. A previous study has shown that the proximal cross-sectional area of the median nerve is weakly correlated with body indices and electrodiagnostic values [17]. In addition, they observed a strong relationship between the proximal and distal area of the carpal tunnel and EMG results [17]. Another study also reported that the proximal and distal cross-sectional areas of the median nerve could be useful for the diagnosis of CTS [18]. However, Ha et al. [19] reported a correlation between EMG results and the cross-sectional areas of the proximal carpal tunnel and the median nerve and no correlation between the carpal tunnel index and EMG results. Also, in our study, the cross-sectional area, AP diameter, and transverse diameter of the median nerve were not correlated with EMG values. The cutoff values used in parameters measured with ultrasound in the carpal tunnel showed differences in the literature [20]. This difference may have been another factor resulting in different outcomes. In this study, we compared US results obtained in the first year after the carpal tunnel release surgery with preoperative US results. The values of the AP diameter and PBD decreased postoperatively, indicating that US can be used in the follow-up of patients after carpal tunnel release surgery.

There is no gold standard procedure for the diagnosis of CTS. The diagnosis of CTS is primarily based on clinical signs and symptoms and correlated with electrodiagnostic results [21]. US is a non-invasive and cost-effective diagnostic method. The diagnosis of CTS using US is based on the change in the median nerve volume and location of the median nerve in the carpal tunnel. There is no gold standard reference for the ultrasonographic diagnosis of CTS [22]. There are many ultrasonographic methods described in the literature [18-20]. Kolovas et al. [22] formed an average ratio based on the evaluation of the AP and transverse diameters of the median nerve in patients with CTS by US. If this ratio was greater than 1.07, it was meaningful, but if it was smaller than 0.79, it was meaningless for CTS. However, they did not investigate a correlation in their study [22]. In this study, we introduced an effective ultrasonographic method for the diagnosis of CTS and analyzed the relationship between our US results and electrodiagnostic findings.

Previous studies used ultrasound for the diagnosis of CTS and compared ultrasonographic findings with EMG results; however, the results were controversial. Mondelli et al. [23] compared the ultrasonographic and electrodiagnostic findings in 70 patients with CTS. Cross-sectional area and the inlet, middle, and outlet of the carpal tunnel were evaluated by ultrasound; according to their results, US was not superior to the electrodiagnostic test for the diagnosis of CTS. Vahed et al. [24] performed EMG and US in 11 patients with the signs and symptoms of CTS. Their study also included 11 patients without the signs and symptoms of CTS in the control group. They reported that the values of the AP and lateral diameters of the carpal tunnel and the median nerve area could be used for the diagnosis of CTS. However, they did not determine the cutoff values. In this study, we determined a high cutoff value and defined a new ultrasonographic method for the diagnosis of CTS.

Patients may have anatomical variations in the carpal tunnel and median nerve [25]. Therefore, the identification of appropriate ultrasonographic parameters is very important for the diagnosis of CTS [13,20]. To minimize the subjectivity associated with the diagnosis, it is very important to use a method in which the anatomical points are clearly identified. Therefore, we recommend the PBD, which can be measured using clearly described anatomic reference points, as a reliable diagnostic criterion for CTS. In our study, the cutoff value of the PBD was correlated with the diagnosis of CTS with high sensitivity and specificity.

There were some limitations in our study. The study included a small number of patients. Nevertheless, patients were diagnosed and included in the study with regard to CTS, both clinically and electrodiagnostically. Another limitation of the study was not identifying patients with the anatomical variants of the carpal tunnel. The inclusion of 20 patients out of 33 in the postoperative follow-up can be considered as another limitation of the study. The evaluation of patients with a similar mean duration of symptoms could also give us reliable results.

US has been suggested to determine the diagnosis of CTS. The PBD promises to be an effective and reliable ultrasonographic measurement method for diagnosing CTS. We believe that the use of US will be more common in clinical practice for diagnosing CTS.

## Conclusions

This study indicates that the ultrasonographic measurement of the PBD is an effective and reliable method for the diagnosis of CTS. We believe that the use of US will be more common in clinical practice for diagnosing CTS.
